# Atypical Hemorrhages Secondary to Dengue Infection and Cardiopulmonary Resuscitation: Subcutaneous Thrombin Injection as a Salvage Technique for Refractory Femoral Artery Hemorrhage

**DOI:** 10.7759/cureus.62581

**Published:** 2024-06-18

**Authors:** Chee W Yap, Kannan Chidambaram, Jun Jie Ng, Genevieve Tan, Shao J Ong

**Affiliations:** 1 Radiology, National University Hospital, Singapore, SGP; 2 Surgery, National University Health System, Singapore, SGP; 3 Advanced Internal Medicine, National University Hospital, Singapore, SGP; 4 Radiology, National University of Singapore, Singapore, SGP

**Keywords:** massive hemorrhage, therapeutic embolization, complications (cpr), thrombin injection, complications of dengue fever

## Abstract

Dengue fever is a viral infection transmitted by the bites of female *Aedes* mosquitoes. Repeat infections with different viral serotypes are possible, with an increased risk of severe dengue. Dengue hemorrhagic fever is one of the most severe presentations of dengue, with thrombocytopenia, increased capillary permeability with resultant rash, and an increased risk of spontaneous bleeding. The management of severe dengue is done through supportive care and symptomatic management only, as there are no specific treatments available. We describe a case of severe dengue hemorrhagic fever presenting with atypical hemorrhage from both the psoas muscle and the femoral arterial puncture sites. These were successfully treated with large calibrated Gelfoam particle embolization for the psoas hemorrhage and regional thrombin injection for the femoral arterial puncture sites.

## Introduction

Dengue fever is a viral infection caused by dengue viruses (Flaviviridae), which are transmitted by infected *Aedes* mosquitoes (*Ae. aegypti* or *Ae. albopictus*) to humans. It is more common in tropical and subtropical regions. The World Health Organization (WHO) documented a 10-fold surge in the dengue cases reported worldwide from 2000 to 2019, increasing from 500,000 to 5.2 million cases [1]. Over 6000 dengue-related deaths were reported in 2023 across 92 countries/territories [2]. The incubation period is approximately four to seven days after infection before clinical manifestation. Up to 75% of dengue infections are asymptomatic. The symptomatic cases may present with fever, headache, retro-orbital pain, musculoskeletal pain, nausea, vomiting, epigastric pain, skin rashes, and bleeding [3]. It is a spectrum of diseases that can be classified into dengue fever without warning signs, dengue fever with warning signs, and severe dengue. The warning signs are severe abdominal pain or tenderness, persistent vomiting, clinical fluid accumulation (i.e., pleural effusion and ascites), mucosal bleeding, lethargy or restlessness, liver enlargement > 2 cm, and laboratory findings of increasing hematocrits with a rapid decrease in platelet counts. The criteria for severe dengue are (one or more of): (i) severe plasma leakage leading to shock, fluid accumulation with respiratory distress; (ii) severe bleeding; and (iii) severe organ involvement (i.e., hepatitis, encephalitis, aseptic meningitis, myocarditis) [4]. The dengue virus has four serotypes: DEN-1, DEN-2, DEN-3, and DEN-4. Infection with one serotype provides long-term immunity to the same serotype. Secondary infection with different serotypes increases the risk of severe dengue [1].

## Case presentation

A 75-year-old female Asian patient presented to the hospital with a left facial droop and associated numbness. The initial investigations, including computed tomography (CT) of the brain and blood work, were unremarkable (including hemoglobin of 11.2 g/dL and platelets of 375 x 10^9^/L). The COVID-19 antigen was negative, and the high-sensitivity C-reactive protein was 12.0 mg/L. The patient developed a fever on day three of admission (temperature: 38.3 °C) with no localizing symptoms. A septic screen was performed, which included a urine test, a chest radiograph, and a repeat COVID antigen test, all of which yielded negative results.

On day six of admission, dengue NS-1 serology (IgM and IgG) was positive. A repeat complete blood count demonstrated a drop in platelet count to 13 x 10^9^/L while hemoglobin levels remained stable at 12.5 g/dL. The patient's antiplatelet medications (aspirin and clopidogrel) were stopped, and the patient was placed on dengue nursing precautions.

On day nine, the patient complained of left hip pain but attributed it to posture. A radiograph of the hip was unremarkable. The patient subsequently had a cardiac arrest episode. Cardiopulmonary resuscitation (CPR) was initiated, and a return of spontaneous circulation was achieved. The resuscitation blood tests demonstrated a drop in hemoglobin levels to 7.1 g/dL. A massive transfusion protocol was activated in view of a likely internal hemorrhage from an unknown source.

**Figure 1:**
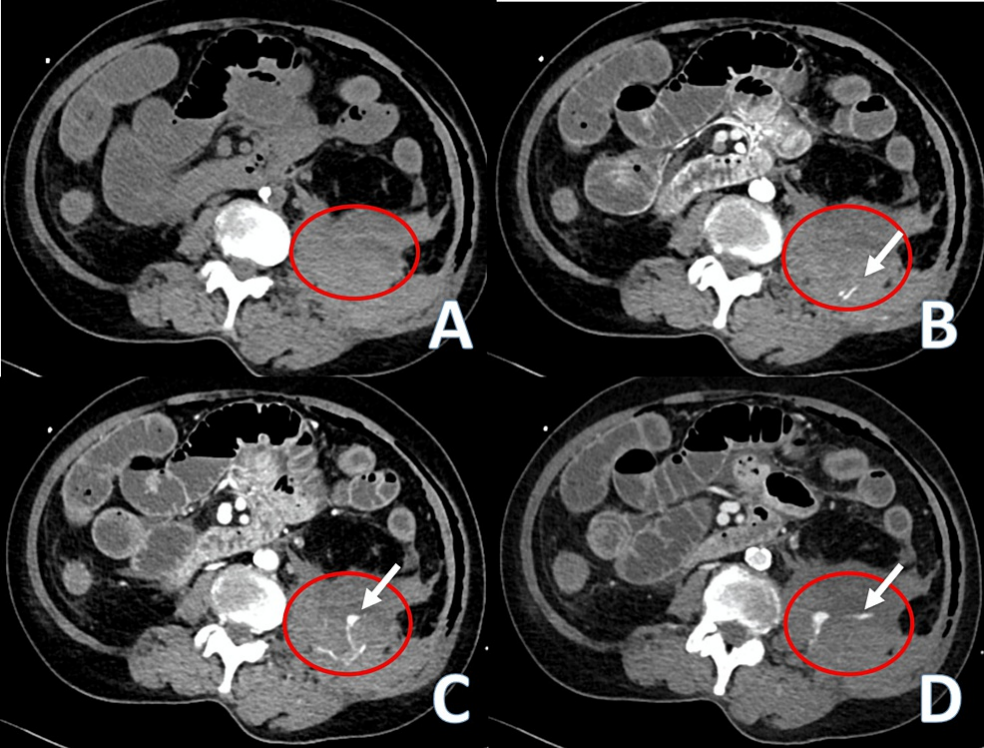
The pre-contrast (A), arterial (B), portal venous (C), and delayed (D) axial CT images showed left retroperitoneal hematoma (red circle), with active contrast extravasation (white arrow) and contrast pooling (white arrow) in the portal venous and delayed phases in keeping with active hemorrhage.

A multiphasic CT scan was performed, revealing a large 10 cm hematoma located in the left retroperitoneal area with active contrast extravasation (Figure 1). The patient was directly transferred from CT to the angiography suites for urgent embolization.

**Figure 2:**
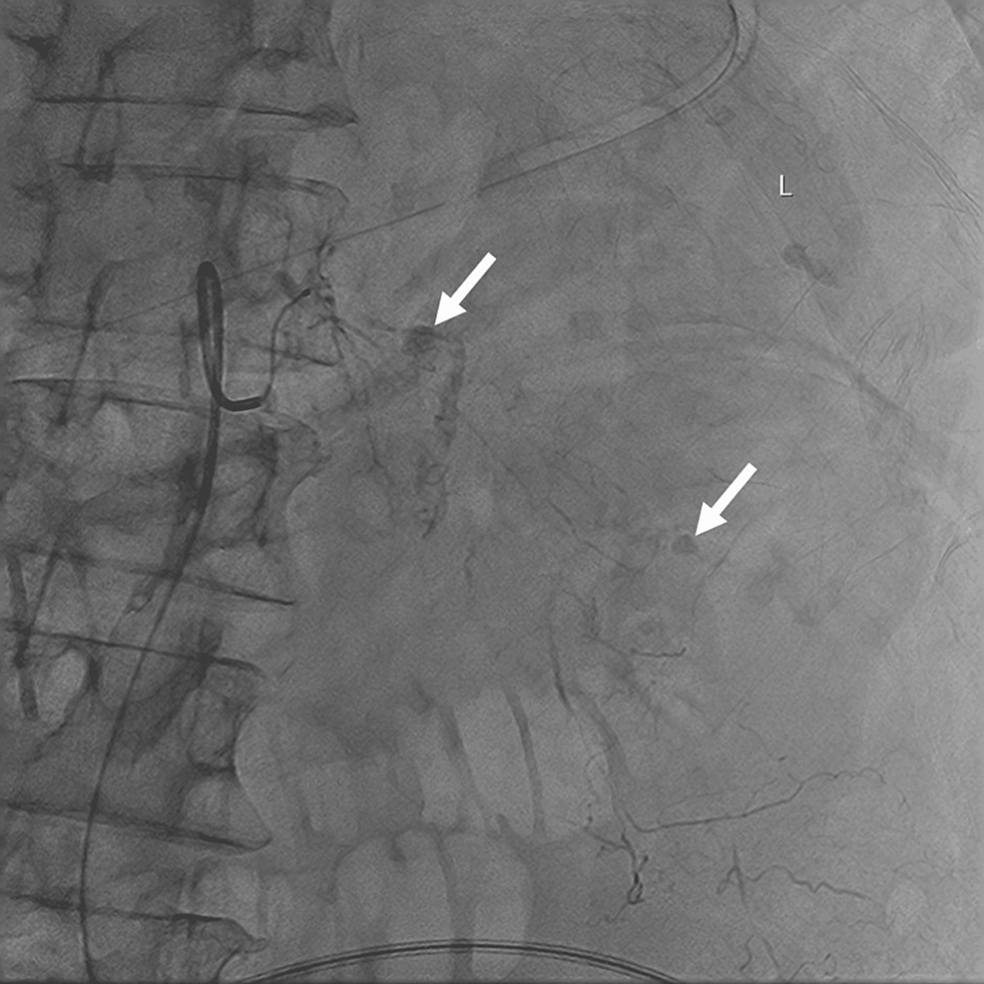
Angiograms via a lumbar artery demonstrated multifocal hemorrhage (white arrows).

Vascular access was obtained by upsizing indwelling left femoral arterial line access obtained during resuscitation rather than attempting a new access site in view of ongoing coagulopathy. Angiograms demonstrated multiple foci of extravasation within the retroperitoneal hematoma (Figure 2). The culprit arteries were embolized with 710-1000 microns calibrated Gelfoam particles (EGgel, ENGAIN, Hwaseong-si, South Korea) with a good final angiographic result.

**Figure 3:**
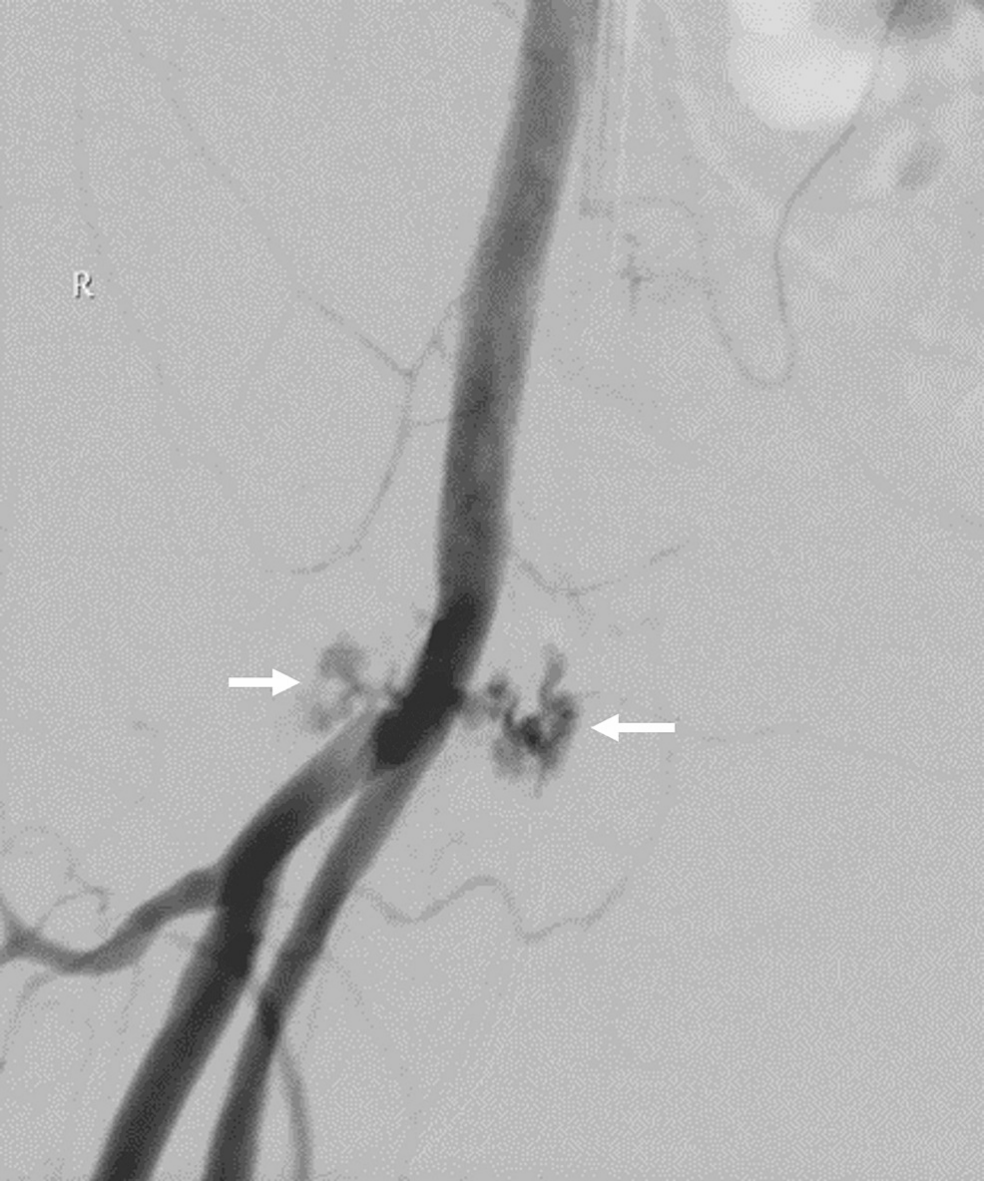
Atypical contrast extravasation (white arrows) arising from the distal right common femoral artery at the level of the bifurcation.

**Figure 4:**
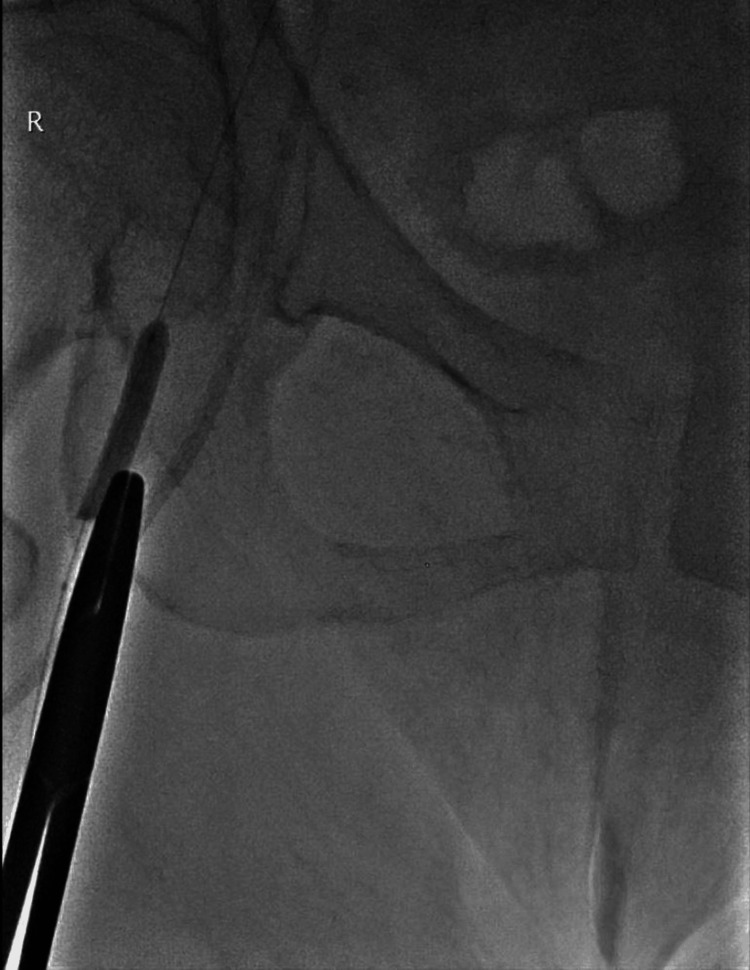
Forceps marking the location of the skin puncture marks. A balloon filled with both contrast medium and air was used to occlude the superficial femoral artery. The forceps was subsequently removed, and manual compression was then performed for 20 minutes in an attempt to obtain hemostasis.

A periprocedural development of a right groin hematoma was noted between the initial CT scan and embolization of the retroperitoneal hematoma. The angiogram of the right common femoral artery (CFA) showed an atypical pattern of hemorrhage at the bifurcation (Figure 3). The appearance did not appear typical for a pseudoaneurysm, and the bleeding did not correspond to any small arterial branch that may have been transacted during the insertion of a central line into the right CFA. On visual inspection, the area of the atypical blush corresponds to needling marks on the skin, likely due to femoral stabs during resuscitation. An initial attempt for hemostasis was performed with a combination of intravascular balloon occlusion and manual compression at the site of the needle puncture marks (Figure 4). However, this was unsuccessful despite prolonged manual compression.

**Figure 5:**
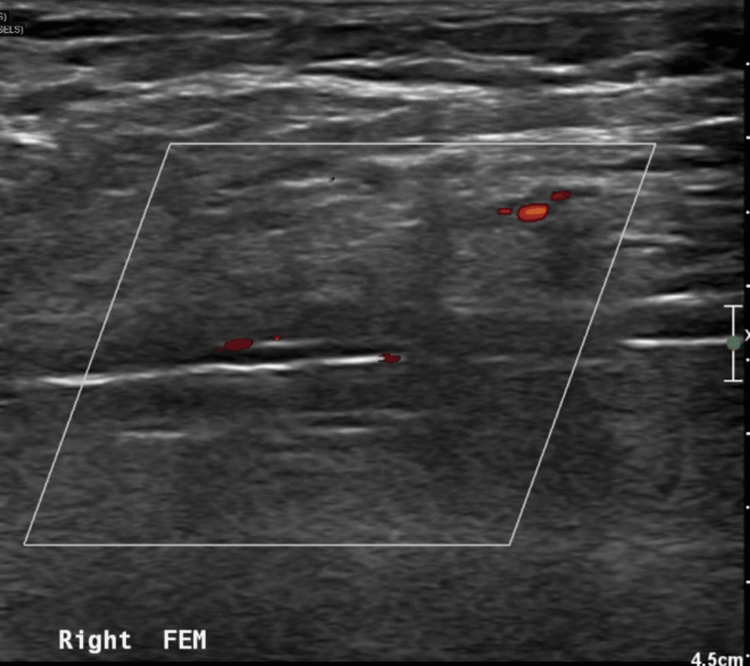
Longitudinal ultrasound image of the right common femoral artery with an inflated balloon in the lumen.

**Figure 6:**
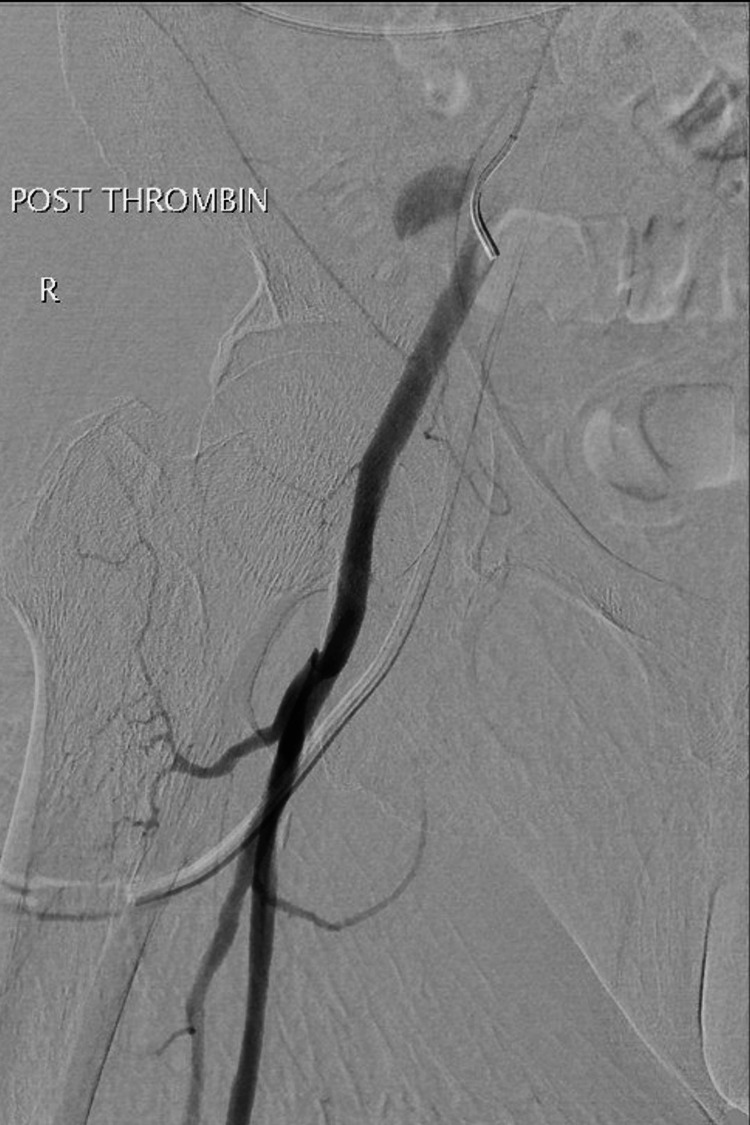
The post-thrombin injection angiogram showed resolution of the atypical arterial blush.

A salvage attempt for hemostasis was performed with a combination of balloon occlusion of the parent vessel while injecting thrombin around the region of the vessel puncture sites. This was performed with the use of a 25G spinal needle with a total of 2000 IU of recombinant thrombin (RECOTHROM, Baxter, Deerfield, Illinois) around the vessel under ultrasound guidance (Figure 5). A good angiographic result was obtained with complete resolution of the atypical hemorrhage after thrombin injection (Figure 6).

## Discussion

Early recognition and monitoring of severe dengue is crucial to reduce dengue morbidity and mortality. The mortality rate of severe dengue fever is approaching 50% if left untreated. This can be decreased to 1% with early, appropriate medical treatment [5]. Severe, life-threatening spontaneous arterial hemorrhage in patients with severe dengue is a rare but known complication (hemoperitoneum, bleeding from the intercostal artery, bleeding from the inferior epigastric artery, and splenic rupture) [5-7].

In this case, the patient's initial assumption that her posture caused her hip pain is likely to have been a precursor to the psoas hemorrhage, which led to the subsequent cardiac arrest. While platelet transfusions are often given to thrombocytopenic dengue patients, there are currently no clear evidence-based guidelines to support blanket platelet-only transfusions without hemorrhagic manifestations [8]. This is likely due to the complexity of the deranged coagulation pathway in dengue infection, where platelets are only one (although much more commonly measured) affected component within the inflammatory process, affecting the coagulation and hemostasis cascade [9].

The interval development of the groin hematoma between the initial CT examination and the completion of the retroperitoneal hematoma embolization is likely to be attributed to the disruption of the normal coagulation cascade. The patient was receiving blood products as per a massive transfusion protocol, which included red blood cells, platelets, and clotting factor concentrates. While these were sufficient together with the calibrated Gelfoam particles to cause occlusive hemostasis within the psoas hematoma, it was insufficient to support manual compression hemostasis on the needle pricks of the CFA caused during cardiopulmonary resuscitation.

Thrombin injection is usually a method of occluding pseudoaneurysms caused by large bore arterial access during endovascular procedures, where thrombin is directly injected into the pseudoaneurysm. In this situation, this was performed around the region of the needle stick access points as there was no clear target lesion, which was a last-ditch attempt to obtain hemostasis. The procedure successfully resolved active hemorrhage and improved the patient's hemodynamics. The patient was subsequently transferred to the intensive care unit for follow-up management.

To the best of the authors' knowledge, this is the first report where hemostasis was successfully obtained by thrombin injection performed in soft tissue surrounding femoral needle stick access points in the CFA with significant hemorrhage refractory to manual compression and balloon occlusion due to dengue infection.

## Conclusions

In cases of dengue hemorrhagic fever, even small-gauge needle arterial access can result in significant arterial hemorrhage in view of the deranged clotting cascade. These may appear as abnormal/atypical blushes on angiograms rather than as pseudoaneurysms, which are more typical of vascular access injuries.

Protective balloon occlusion with targeted regional thrombin injection is a potential salvage procedure to obtain hemostasis in such cases.
